# Psychoactive pollutant alters movement dynamics of fish in a natural lake system

**DOI:** 10.1098/rspb.2024.1760

**Published:** 2024-12-11

**Authors:** Jack A. Brand, Michael G. Bertram, Daniel Cerveny, Erin S. McCallum, Gustav Hellström, Marcus Michelangeli, Daniel Palm, Tomas Brodin

**Affiliations:** ^1^ Department of Wildlife, Fish, and Environmental Studies, Swedish University of Agricultural Sciences, Umeå 907 36, Sweden; ^2^ Institute of Zoology, Zoological Society of London, London NW1 4RY, UK; ^3^ School of Biological Sciences, Monash University, Clayton, Victoria 3800, Australia; ^4^ Department of Zoology, Stockholm University, Stockholm 114 18, Sweden; ^5^ University of South Bohemia in Ceske Budejovice, Faculty of Fisheries and Protection of Waters, South Bohemian Research Center of Aquaculture and Biodiversity of Hydrocenoses, Zatisi 728/II, Vodnany, Czech Republic; ^6^ Australian Rivers Institute, Griffith University, Nathan, Queensland 4111, Australia

**Keywords:** behaviour, chemical, ecology, global change, survival, telemetry

## Abstract

Pharmaceutical pollution poses an increasing threat to global wildlife populations. Psychoactive pharmaceutical pollutants (e.g. antidepressants, anxiolytics) are a distinctive concern owing to their ability to act on neural pathways that mediate fitness-related behavioural traits. However, despite increasing research efforts, very little is known about how these drugs might influence the behaviour and survival of species in the wild. Here, we capitalize on the development of novel slow-release pharmaceutical implants and acoustic telemetry tracking tools to reveal that exposure to environmentally relevant concentrations of the benzodiazepine pollutant temazepam alters movement dynamics and decreases the migration success of brown trout (*Salmo trutta*) smolts in a natural lake system. This effect was potentially owing to temazepam-exposed fish suffering increased predation compared with unexposed conspecifics, particularly at the river–lake confluence. These findings underscore the ability of pharmaceutical pollution to alter key fitness-related behavioural traits under natural conditions, with likely negative impacts on the health and persistence of wildlife populations.

## 1. Introduction

Pharmaceutical pollution is a major threat to human health and global biodiversity [1–3]. Indeed, more than 900 different active pharmaceutical ingredients have now been detected in aquatic ecosystems worldwide [4,5]. These contaminants often target evolutionarily conserved biological pathways and are designed to be effective at low concentrations [4,6], highlighting their potential to affect wildlife. Further, with the pharmaceutical industry predicted to grow by 3−6% annually [7], this problem is only expected to worsen into the future. Thus, understanding how species are affected by pharmaceutical pollution is crucial to ongoing environmental protection efforts.

Research over the past several decades has shown that high concentrations of pharmaceutical pollutants can influence wildlife development, reproduction and survival [8–10]. However, more recent work has emphasized that even low, environmentally realistic pharmaceutical concentrations can have pervasive sub-lethal effects on physiology and behaviour, which may alter individual- and population-level fitness [6,11]. In this regard, psychoactive pharmaceutical pollutants (e.g. anti-depressants, anxiolytics, painkillers) pose an especial risk owing to their ability to act on neural pathways that alter fitness-related behavioural traits [6,11,12]. Psychoactive pollutants have repeatedly been detected in the tissues of wild organisms—including in the brain [13,14]—at concentrations known to elicit behavioural effects (i.e. ng g^−1^) [15–17], emphasizing the potential for these pollutants to have substantial ecological and evolutionary impacts.

Most of the current evidence for the effects of psychoactive pharmaceuticals on the behaviour of wildlife has investigated animal behaviour under standardized laboratory conditions [11,15,18]. Given that behavioural traits expressed in the laboratory are often not representative of those expressed in the wild [19–21], understanding how exposure to psychoactive pollutants alters behaviour in natural settings is imperative. Moreover, whether pharmaceutical-induced behavioural alterations have fitness-related consequences for wild organisms is not well-known. Thus, there is an urgent need for field-based experimental research to investigate whether pharmaceutical contaminants affect the behaviour and fitness of wildlife under ecologically realistic conditions.

**Figure 1.**
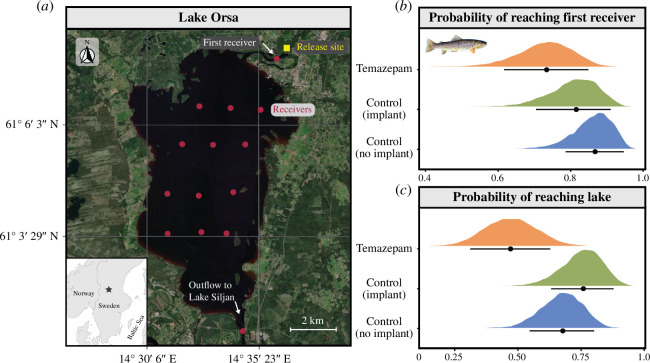
(*a*) Overview of Lake Orsa. The yellow square denotes the release site in the River Ore, while the red circles indicate the position of each of the 14 acoustic receivers. The location of Lake Orsa in central Sweden is indicated by the black star on the grey map insert. Result plots show the probability of reaching the (*b*) first receiver in the array, located in the River Ore (i.e. *ca* 650 m downstream of the release site) and (*c*) any receiver in Lake Orsa or the downstream outflow in the temazepam (orange), control–implant (green) and control–no implant (blue) treatment groups. Coloured distributions display the respective posterior distributions extracted from Bayesian generalized linear models, while point estimates and error bars represent the posterior median and 89% credible intervals. The map was sourced from ESRI World Imagery. Note: the *x*-axes in plots (*b*) and (*c*) have been restricted to better visualize treatment comparisons. Trout photo insert credit: Jörgen Wiklund.

Here, we conducted a large, field-based experimental study in Lake Orsa (surface area = 52 km^2^; maximum depth = 94 m)—located in central Sweden (figure 1*a*)—to investigate how psychoactive pharmaceutical pollutants influence the behaviour of brown trout (*Salmo trutta*) in the wild. Brown trout are a salmonid native to freshwater and coastal marine waterways of Europe, western Asia and northern Africa [22]. Despite the species being globally introduced for recreational fishing [23] and listed as ‘Least Concern’ on the IUCN Red List of Threatened Species, local populations of native brown trout have suffered declines in recent years—a trend that is partly attributed to chemical pollution [24,25]. Lake Orsa contains natural populations of adfluvial brown trout that spawn and develop upstream in tributary rivers before migrating to the lake, where they grow and mature. Thus, movement between the river and lake system represents a significant event that is vital to the life-history and fitness of this population. Given that brown trout are considered a species of high ecological and socio-economic value [25], there is a need to understand how pharmaceutical pollution may influence the movement dynamics and subsequent fitness of this species—and other vulnerable fish populations more generally—in the wild.

Prior research has shown that exposure to environmentally realistic concentrations of benzodiazepine pharmaceuticals—a common class of gamma-aminobutyric acid (GABA) agonists that reduce neural activity and are often prescribed for mood and sleep disorders [26]—can affect fish behaviour in the laboratory [12,15], including in brown trout [27]. For example, European perch (*Perca fluviatilis*) exposed to environmental levels of the benzodiazepine drug oxazepam (1.8 µg l^−1^) demonstrated higher activity, increased foraging and reduced social behaviour when compared with controls [12]. However, whether such effects have fitness-related consequences in the wild is less clear. Recent work has demonstrated that exposure to dilute concentrations of the benzodiazepines temazepam (0.08−1.5 µg l^−1^) or oxazepam (1.9 µg l^−1^) can affect short-distance (i.e. *ca* 100 m) migration intensity in sea-run brown trout [16] and Atlantic salmon (*Salmo salar*) [28], respectively. In the case of Atlantic salmon, exposure to high concentrations of oxazepam (i.e. 200 µg l^−1^) has also been shown to increase predation risk in the wild—an effect that was suggested to be owing to pollutant-induced behavioural changes [29]. However, whether exposure to psychoactive pharmaceuticals at environmentally realistic concentrations in the wild affects large-scale movement dynamics over greater distances, with consequences for organismal fitness, is not clear.

We experimentally exposed hatchery-reared brown trout smolts to the benzodiazepine temazepam in the field, using previously validated slow-release chemical implants [30,31]. Temazepam, along with its biologically active metabolite oxazepam, are often individually prescribed to treat insomnia and anxiety-related disorders and are commonly detected in European waterways [4,32,33]. For example, previous analysis of surface waters from European rivers has recorded maximum concentrations of 1.38 µg l^−1^ [4] and 61 ng l^−1^ [32] of temazepam and oxazepam, respectively. After chemical implantation and tagging, all experimental fish in the current study were released into the River Ore, *ca* 2 km upstream of the inlet to Lake Orsa (figure 1*a*), where their movements were tracked using an acoustic telemetry array. Chemical analysis of water samples taken from the River Ore and Lake Orsa confirmed that the sites were free from temazepam and oxazepam pollution (see electronic supplementary material, data 1). Further, an additional laboratory-based study was also conducted to confirm the uptake of temazepam from the chemical implants over the study period, and its biotransformation, by measuring the concentrations of temazepam and its metabolite oxazepam in the tissues of exposed brown trout smolts. Given that previous research has found that exposure to environmentally relevant benzodiazepine concentrations can increase activity rates and risk-taking behaviour in fish under laboratory conditions [6,15,34], as well as increase short-distance migration intensity in brown trout [16] and Atlantic salmon [28] smolts, we expected temazepam-exposed brown trout in our field study to decrease their initial time spent in the River Ore (i.e. near the release site) before migrating to Lake Orsa, relative to control groups. However, such behavioural effects are also expected to increase predation risk [29], and we therefore expected that fewer trout in the temazepam group would ultimately reach Lake Orsa, relative to controls.

## 2. Material and methods

### (a) Study site

The study was conducted in Lake Orsa—a sub basin of Lake Siljan—located in central Sweden (figure 1*a*). Lake Orsa is a large lake with one major inflow that has been regulated for hydropower: the River Ore, with a mean flow rate of 22 m^3^ s^−1^. Lake Orsa contains natural populations of adfluvial brown trout that spawn and develop upstream in tributary rivers before migrating to the lake, where they grow and mature [35]. However, as the major inflow river is regulated for hydropower (dam with no fish passage located *ca* 1.4 km upstream of the release site), a supplementary stocking programe of brown trout smolts has been conducted for several decades [35]. In addition to trout, the fish fauna in the lake largely consists of northern pike (*Esox lucius*), European perch, burbot (*Lota lota*), bream (*Abramis brama*), ide (*Leuciscus idus*), roach (*Rutilus rutilus*) and whitefish (*Coregonus* sp.).

### (b) Slow-release chemical implants

Internal, slow-release chemical implants are a recently developed tool in experimental aquatic ecotoxicology that contain a known concentration of a target chemical suspended in a fat-based carrier [30]. These implants allow the sustained exposure of aquatic organisms to environmentally relevant concentrations of a contaminant of interest in the field [30]. All implants in the current study were prepared following previously established methods [30,31]. In brief, implants were prepared by dissolving temazepam (CAS: 846-50-4; Sigma-Aldrich, Steinheim, Germany) in liquefied coconut oil (Kung’s Markatta Virgin Coconut Oil) to reach a desired concentration of 50 µg of temazepam per gram of implant. The solution was continuously stirred for 10 min to ensure sufficient mixing, before being sonicated in an ultrasound bath for a further 15 min at 30°C [30]. Control implants (i.e. coconut oil without temazepam) were prepared following exactly the same procedure, with the exception that temazepam was not included.

### (c) Fish tagging and release

All experimental procedures were approved by the Swedish Board of Agriculture (permit numbers: Dnr A.18.15 and Dnr 5.8.18). Two-year-old, hatchery-produced brown trout smolts (*n* = 90; body mass [mean ± s.e.] = 96.40 ± 1.58 g) were haphazardly selected from stocks held at the catchment-specific rearing facility in 2020. Three days before their release into the River Ore, smolts were anaesthetized in a tricaine methanesulfonate solution (MS-222; 0.15 g l^−1^; Sigma Aldrich, Steinheim, Germany), weighed and measured (total length), before a small incision (*ca* 20 mm) was made into the abdomen on the ventral surface of the fish. The gills of each fish were kept constantly submerged in clean water throughout the minor surgical procedure. Fish were implanted with an Innovasea V7 69 kHz acoustic tag (weight in air = 1.4 g; dB = 136; Innovasea Systems Inc. Halifax, NS, Canada) with an estimated maximum lifespan of 268 days, as well as a passive integrated transponder (PIT) tag (APT 12 mm tags; Biomark, Idaho, USA) that allowed for the identification of individual fish throughout the release. The transmission rate of acoustic tags was programmed to emit signals every 100 s (randomly varying between 60 and 140 s) for the first 14 days, decreasing to every 160 s (randomly varying between 120 and 200 s) thereafter to maximize battery life. Acoustic tag implantation resulted in a tag burden (tag mass relative to body mass) of *ca* 1.49% (± 0.02%), which is below the recommended upper tag burden limits of 2−10% [36]. Fish were also given the slow-release implant at the same time that each fish was tagged, so that fish were not anaesthetized twice. Sixty randomly chosen fish were implanted with a control implant (*n* = 30) or slow-release chemical implant (i.e. temazepam; *n* = 30) using a blunted 18-gague needle at a dose of 5 µg of implant per gram of body mass in the same incision used for the transmitter insertion, in line with established protocols [30]. The remaining 30 fish received no control or slow-release chemical implant (i.e. they only received acoustic and PIT tags) to account for any potential effects of the slow-release implant itself on fish movement. This resulted in three treatment groups: temazepam (50 µg g^−1^ of temazepam implant, as well as acoustic tag and PIT tag), control with implant (i.e. coconut oil implant without temazepam, as well as acoustic tag and PIT tag) and control with no implant (i.e. only acoustic tag and PIT tags). This careful experimental design with two control groups allowed us to separate the effects of the implant itself on trout behaviour (control–no implant versus control–implant comparisons) from the effects of temazepam alone (control–implant versus temazepam implant comparisons). The incision was then closed using two, non-absorbable silk sutures (Ethicon EH7149G). Following the procedure, fish were returned to holding tanks, where they were left to recover for 3 days. Previous research has found that MS-222 is eliminated rapidly from the plasma of exposed fish (elimination half-life of *ca* 1.7 min in salmonids [37]) and has no observable effect on fish activity or risk-taking behaviour up to 60 min post-recovery [38]. Similar work reported that barramundi (*Lates calcarifer*) exposed to MS-222 (0.09 g l^−1^) achieved a healthy recovery after 3 days, with MS-222 concentrations in the muscle and liver being below the limit of detection 72 h after administration [39]. Thus, the 3-day recovery period ensured there would be minimal effects of MS-222 administration on trout behaviour once released into the wild.

Three days after tagging, fish were released into the River Ore, *ca* 2 km upstream of the inlet to Lake Orsa (figure 1*a*). The release site was also located approximately 1.4 km downstream of Hansjö hydroelectric power plant, which restricted the ability of fish to move further upstream (i.e. a complete blockage without fish passage). All fish were released simultaneously in the same batch at 08:00 h on 22 May 2020. Alongside experimental fish, *ca* 5000 untagged and unexposed brown trout smolts were also released as a part of standard stocking efforts and to reduce potential immediate predation pressure on experimental fish.

### (d) Fish tracking

Twelve acoustic receivers (WR2, Innovasea Systems Inc. Halifax, NS, Canada) were deployed throughout Lake Orsa (figure 1*a*) in order to track fish equipped with acoustic tags. One additional receiver was placed at the inlet (River Ore) of the lake, *ca* 650 m downstream of the release site. Similarly, a receiver was also placed at the outlet where Lake Orsa flows into Lake Siljan (electronic supplementary material, table S1). The receivers were attached to buoys to ensure that they were maintained in a vertical position in the water column, and were secured with weights *ca* 3−5 m above the bottom of the lake. Receivers were deployed on 1 May 2020 and retrieved throughout May 2021.

### (e) Collection of water samples for chemical analysis

Water samples were collected from the study site for broad-spectrum chemical analysis to characterize the profile of pharmaceutical pollution in the area—considering that pharmaceuticals are present, at least at trace concentrations, in essentially all human-impacted ecosystems [4,40]. Specifically, a surface water sample (*ca* 150 ml) was collected from the release site (e.g. the River Ore) on the day of the release. Similarly, water samples (*n* = 3) were taken every three months in Lake Orsa (61°04'12.1' N, 14°30'04.2' E) during the initial six months of the tracking study (i.e. until November 2020 and before lake ice prohibited the collection of surface water samples). After collection, water samples were immediately stored at −20°C until extraction and subsequent analysis (see §2g).

### (f) Confirmation of pharmaceutical uptake in fish

Alongside the field-based tracking study, we performed a laboratory experiment to confirm the uptake and biotransformation of temazepam in brown trout tissue from the slow-release chemical implants. Forty-one, one-year-old brown trout smolts (body mass [mean ± s.e.] = 30.58 ± 2.52 g) that were collected from the Norrfors Vattenfall Fish Hatchery on the River Ume (63°52'45' N, 20°01'06' E) were implanted with slow-release chemical implants containing temazepam following exactly the same procedures as used in the field study (but without any acoustic tag). Fish were housed in one of two tanks that were constantly supplied with natural river water. Starting three days after implanting, *ca* 3 fish (range = 1−5) were humanely euthanized in an MS-222 solution (0.3 g l^−1^) and immediately frozen at −20°C for later chemical analysis. This process was repeated at 12 different timepoints between 3 and 154 days post implanting (electronic supplementary material, tables S2 and S3). In May 2021, these fish were dissected for brain, liver and muscle samples to investigate the concentration of both temazepam and its biologically active metabolite oxazepam in these tissues.

### (g) Preparation of samples and instrumental analysis

Water samples collected from experimental sites were extracted by solid phase extraction (SPE) using 6 cc Oasis HLB cartridges containing 200 mg of sorbent (Waters, Milford, MA). The cartridges had first been conditioned with 5 ml of HPLC-grade methanol (Merc, Darmstadt, Germany), followed by 5 ml of ultrapure water (Milli-Q). Then, 150 ml of the water sample were spiked with a mixture of 14 isotopically labelled internal standards and passed through the cartridge at a pace of approximately 1 ml min^−1^ using a vacuum manifold (VacMaster, Biotage, Uppsala, Sweden). The target analytes were eluted from the sorbent using 5 ml of HPLC-grade methanol, followed by 5 ml of HPLC-grade ethyl acetate, and the eluent was captured in a 12 ml glass vial and subsequently evaporated to dryness using a TurboVap LV (Biotage, Uppsala, Sweden). Each sample was then reconstituted with 150 µl of LC-MS-grade methanol (Merc, Darmstadt, Germany), transferred into a 1.5 ml autosampler vial equipped with a 200 µl glass insert and stored at −20°C until analysis. Details about the target compounds, together with their limits of quantification (LOQs), are provided in the electronic supplementary material, table S4.

The tissue samples were pre-treated following previously described protocols [15]. In short, samples underwent repeated solvent extraction using HPLC-grade acetonitrile (Merc, Darmstadt, Germany) acidified with LC-MS-grade formic acid (Merc, Darmstadt, Germany) to 0.1%. The supernatant from both extractions was combined, evaporated and reconstituted with 150 µl of LC-MS-grade methanol.

All samples were analysed using liquid chromatography-tandem mass spectroscopy (LC-MS/MS). We used a triple-stage quadrupole mass spectrometer (TSQ Quantiva, Thermo Scientific, San José, CA, USA) equipped with a heated-electrospray ionization (HESI) ion source to analyse both SPE-extracted water samples and tissue extracts. The instrument was coupled to an Accela LC pump (Thermo Fisher Scientific, San José, CA, USA) and a PAL HTC autosampler (CTC Analytics AG, Zwingen, Switzerland). A C18 phase Hypersil gold column (50 × 2.1 mm ID × 3 µm particles, Thermo Fisher Scientific, San José, CA, USA) was used for liquid chromatography to separate the target analytes before mass spectrometry analysis. Ultrapure water was prepared in-house using a Millipore purification system (Merc, Darmstadt, Germany) and LC-MS-grade methanol, both of which were acidified with formic acid at 0.1% and were used as a mobile phase during the LC-MS/MS analysis.

Linearity, precision, limit of quantification (LOQ) and measurement of blank samples were used as the quality assurance and quality control (QA/QC) for the analytical methods used in this study. Quantification of target compounds was carried out using isotopic dilution (the internal standard approach). Instrumental LOQ was derived from a six-point standard curve from 0.1 to 50 ng g^−1^. Peak area corresponding to the lowest point of the calibration curve that had a signal : noise ratio of at least 10 was then used for calculation of LOQs in individual samples. Precision was expressed as a relative standard deviation (RSD) of response factors calculated for each point of the calibration curve. Tissue samples that were < LOQ were given half the relevant LOQ for inclusion in mean concentration calculations, in line with previous research [30]. Caffeine was the only target analyte detected in blank samples prepared during the SPE extraction of water samples. The concentration of caffeine in blanks ranged between 10.8 and 11.1 ng l^−1^, with the latter having been subtracted from all other water samples analysed in the study. Results from the water sample analysis showed that, apart from caffeine and two samples where budesonide (corticosteroid prescribed for asthma) was detected (7.72−12.79 ng l^−1^), water samples from the study site were free from pharmaceutical contamination (see electronic supplementary material, data 1).

### (h) Data analysis

All data analysis was performed in R (v. 4.2.2) [41]. Initial data filtering for false detections was performed using the *ATfiltR* [42] and *tidyverse* [43] packages. We first removed detections from individual fish that were recorded only once within a 1 h time window at a given receiver, in accordance with previously established methods [44]. Where simultaneous detections were recorded for an individual at a given receiver, we removed one of the observations. We inspected abacus plots for each individual in the dataset. Where detections for an individual were constant (i.e. it no longer moved between receivers), frequent and uniform without any changes for a prolonged period at a given receiver (e.g. weeks to months), we deemed this individual to have died and removed these observations from the analysis (*n* = 4 individuals).

Similar to previous studies on fish movement and behaviour (e.g. [45,46]), we employed Bayesian (generalized) linear models for all analysis using the *brms* package [47]. *Post hoc* comparisons between treatment groups were executed using the *emmeans* [48] package and the *modelbased* package from the *easystats* suite [49]. All models were run for 3000 iterations with 1000 warmup iterations, across four chains and with weakly informative priors, as suggested by Lemoine [50]. To ensure adequate model fits, we conducted posterior predictive checks, and the examination of trace plots and the Gelman–Rubin diagnostic statistic indicated that models had converged with minimal among-chain variability ($\hat{R}=1.00$). We present posterior medians with 89% highest posterior density credible intervals (CI) for all parameter estimates, in line with previous recommendations due to their increased stability over 95% CI at lower effective sample sizes [51,52]. Where appropriate, body mass was scaled and mean-centred (mean = 0; s.d. = 1) before being included as a covariate in models, allowing us to estimate effects for an average-sized fish.

#### (i) Lake migration success and total time detected

We investigated the probability of fish successfully reaching the first receiver in the River Ore using a Bayesian generalized linear model with a Bernoulli distribution (logit link). Detection success at the first receiver (1 or 0) was included as a binary response variable, while treatment was included as a fixed-effect factor and body mass (scaled) as a continuous covariate. Additionally, for those fish that were successfully detected at the first receiver, we investigated the probability of subsequently reaching Lake Orsa (i.e. being detected on any receiver located in Lake Orsa or at the outlet point to Lake Siljan). The model structure was exactly the same as described above, except that lake success (1 or 0) was included as the response variable.

To further explore whether potential treatment differences in the probability of successfully reaching Lake Orsa were owing to differences in the time spent in the river, we analysed the total time that fish initially spent in the River Ore using a Bayesian generalized linear model with a gamma distribution (log link). Specifically, we took the time difference (in days) between a fish’s first and last detections on the receiver located in the River Ore. Given that four fish returned to the river after visiting the lake during the study period, we restricted analysis to include only the initial time that these fish spent in the river before entering the lake. Total time initially spent in the river was included as a response variable, while treatment was included as a fixed-effect factor and body mass (scaled) as a continuous covariate. Similarly, whether fish successfully entered Lake Orsa (1 or 0) was also included as a covariate in the model, which controlled for any potential differences in the initial time spent in the river between fish that did and did not successfully reach the lake.

We also explored whether exposure to temazepam influenced the total time that fish were detected in the receiver array using a Bayesian generalized linear model with a gamma distribution (log link). The total time that fish were detected in days (i.e. time elapsed between release date and final detection) was used as a response variable. Fish that were released but were not subsequently detected on any receiver (*n* = 18) were assigned a value of 0.001 days (i.e. *ca* 1 min), approximately corresponding to the time during which these fish were observed during release. Treatment was included as a fixed-effect factor, while body mass (scaled) was included as a continuous covariate.

#### (ii) Movement and space use

**Figure 2.**
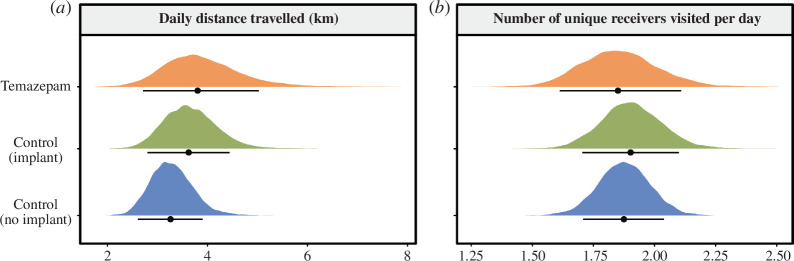
Result plots show the (*a*) daily distance travelled (km) and (*b*) number of unique receivers visited per day by each fish during the study period in the temazepam (orange), contro–implant (green) and contro–no implant (blue) treatment groups. Coloured distributions display the respective posterior distributions extracted from the Bayesian linear models, while point estimates and error bars represent the posterior median and 89% credible intervals, respectively.

We calculated the movement and space use of those fish that successfully reached Lake Orsa (figure 2). We used a shapefile of our study site to generate a transition matrix in order to estimate the daily in-water distance travelled for all fish that successfully reached Lake Orsa, using the *shortestPath*() function from the *gdistance* package [53]. We restricted analysis to the first 97 days of exposure (i.e. 93 days of tracking) because the laboratory experiment indicated that 100% of brown trout brain, liver and muscle tissue samples were below the LOQ for temazepam and oxazepam after this timepoint. Previous research in Atlantic salmon smolts found that fish moved a maximum of *ca* 15.6 km per day through lakes [54,55]. We therefore excluded any daily distance estimates > 16 km in our analysis. Daily distance travelled (in km) during the study was included as a response variable in a Bayesian generalized linear model with an exponential distribution (log link). Treatment was included as a fixed-effect factor, while body mass (scaled) and Julian day (scaled) were included as continuous covariates. Fish ID was included as a random intercept to control for repeated measures. As overlapping detection ranges of receivers may influence estimates of distance travelled, we also performed an additional supplementary pseudo-position-based analysis [56]. Here, we took the average longitude and latitude of fish detections during 30 min intervals to calculate ‘centres of activity’ [56]. The in-water distance travelled between subsequent pseudo-positions was then calculated as outlined above. Such an approach reduces the chance of including rapid subsequent detections at two overlapping receivers in estimates of distances travelled. The results from this analysis (electronic supplementary material, table S11) were qualitatively similar to those reported in the main text.

For these same fish, we also calculated the total number of unique receivers visited by each individual per day during the initial 97 days of exposure (i.e. 93 days of tracking). The number of unique receivers visited by each fish per day was included as a response variable in a Bayesian generalized linear mixed-effects model with a Poisson distribution (log link). Treatment was included as a fixed-effect factor, while body mass (scaled) and Julian day (scaled) were included as continuous covariates. Fish ID was also included as a random intercept to account for repeated measures.

## 3. Results

### (a) Lake migration success and total time detected

Temazepam exposure decreased the probability of successfully reaching the first receiver in the River Ore (located *ca* 650 m downstream of the release site; figure 1*b*). Specifically, the probability of reaching the first receiver was lower in the temazepam-exposed group (probability [89% CI] = 0.73 [0.62, 0.85]; 21 successful fish) when compared with the control–no implant group (probability [89% CI] = 0.87 [0.79, 0.95]; 27 successful fish; figure 1*b*; electronic supplementary material, table S5). However, there was no clear difference between the temazepam and control–implant groups (probability [89% CI] = 0.82 [0.71, 0.91]; 24 successful fish), nor between either of the control groups (figure 1*b*; electronic supplementary material, table S5). Body mass had no substantial effect on the probability of reaching the first receiver (estimate [89% CI] = −0.06 [−0.48, 0.36]).

For fish that were detected by the first receiver, temazepam exposure also reduced their probability of subsequently reaching Lake Orsa (*ca* 4.5 km from the release site to the initial array of receivers within the lake). In particular, temazepam-exposed fish had a decreased probability of reaching Lake Orsa (probability [89% CI] = 0.47 [0.32, 0.64]; 9 successful fish) compared with both the control–no implant (probability [89% CI] = 0.68 [0.55, 0.80]; 19 successful fish) and control–implant (probability [89% CI] = 0.76 [0.63, 0.88]; 18 successful fish) groups (figure 1*c*; electronic supplementary material, table S6). We also found a marginally positive effect of body mass on the probability of reaching the lake, with larger fish being slightly more likely to reach the lake than their smaller counterparts (estimate [89% CI] = 0.38 [−0.04, 0.85]), although there was substantial uncertainty around this effect. Only two fish (1 control–no implant and 1 control–implant) were detected by the receiver in the outlet where Lake Orsa flows into the larger Lake Siljan (*ca* 15.1 km from the release site).

Control–no implant fish also initially spent less time within the detection range of the receiver in the River Ore (median [89% CI] = 1.46 days [0.72, 2.41]), compared with both the control–implant (median [89% CI] = 3.96 days [2.09, 6.32]) and temazepam-exposed (median [89% CI] = 3.30 days [1.62, 5.37]) treatment groups (electronic supplementary material, table S7). There were no differences between the temazepam-exposed and control–implant treatment groups in this regard (electronic supplementary material, table S7). Body mass influenced the initial time spent in the river, with larger fish spending a longer period of time in the river (estimate [89% CI] = 0.51 [0.12, 0.95]).

We found no evidence of a treatment effect on the total time that fish were detected in the array, with no substantial differences in total detection time between fish from the temazepam (median [89% CI] = 46.32 days [20.77, 78.15]), control–implant (median [89% CI] = 47.68 days [21.23, 81.55]) and control–no implant (median [89% CI] = 54.57 days [26.95, 88.82]) treatment groups (electronic supplementary material, table S8). However, we found a weakly positive effect of fish body mass on the total time detected, with larger fish being detected for marginally longer than their smaller counterparts (estimate [89% CI] = 0.27 [−0.08, 0.66]), although with considerable uncertainty around the estimate.

### (b) Movement and space use

After controlling for body mass and Julian day, there were no differences in the estimated daily distance travelled by each fish during the study period between the temazepam (median [89% CI] = 3.81 km [2.69, 5.00]), control–implant (median [89% CI] = 3.63 km [2.87, 4.50]) or control–no implant (median [89% CI] = 3.26 km [2.58, 3.88]) treatment groups (figure 2*a*; electronic supplementary material, table S9). However, there was a positive effect of body mass (estimate [89% CI] = 0.17 [0.02, 0.31]) and a negative effect of Julian day (estimate [89% CI] = −0.24 [−0.31,−0.16]) on the daily distance travelled by trout—i.e. regardless of treatment, larger fish travelled further distances and fish travelled shorter daily distances as time in the study progressed.

There were no differences in the number of unique receivers that each fish visited per day between the temazepam (median [89% CI] = 1.85 [1.60, 2.10]), control–implant (median [89% CI] = 1.90 [1.70, 2.10]) or control–no implant (median [89% CI] = 1.87 [1.71, 2.04]) treatment groups (figure 2*b*; electronic supplementary material, table S10). While there was no effect of body mass (estimate [89% CI] = 0.02 [−0.04, 0.08]), there was a negative effect of Julian day on the number of unique receivers that each fish visited per day (estimate [89% CI] = −0.07 [−0.12,−0.03]).

### (c) Chemical uptake in fish tissues

**Figure 3.**
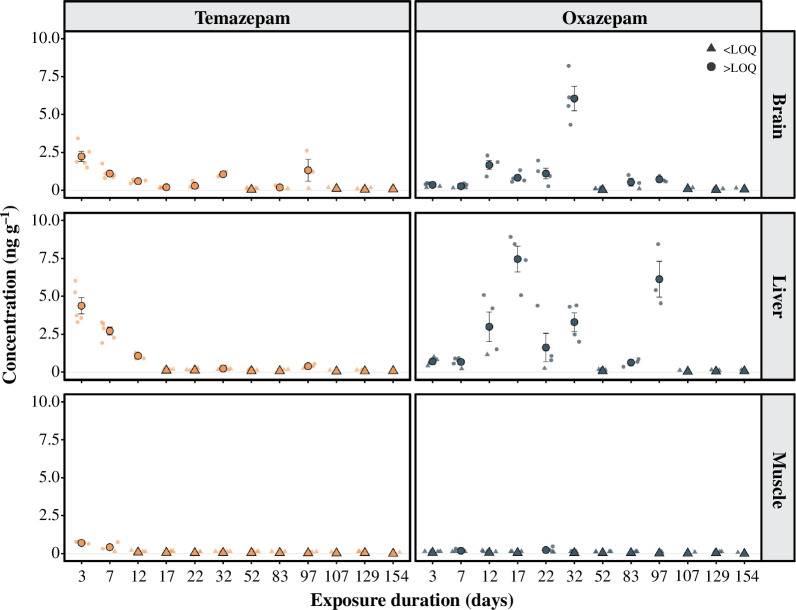
Concentrations (ng g^−1^) of temazepam (orange) and its biologically active metabolite oxazepam (dark blue) in the brain, liver and muscle of exposed fish from the laboratory study. Point estimates represent mean concentrations, while error bars denote ± 1 s.e. Small, semi-transparent circle data points represent observations that were above the limit of quantification (LOQ), while small, semi-transparent triangle data points indicate observations that were below the LOQ. These latter observations were given half the relevant LOQ for inclusion in concentration calculations, in line with previous research [30]. Mean estimates for a given exposure duration where all samples were below LOQ are also indicated by triangle point estimates. LOQ mean ± s.e.; temazepam = 0.383 ± 0.028 ng g^−1^; oxazepam = 0.351 ± 0.025 ng g^−1^.

Results from the laboratory study indicated that tissue concentrations of temazepam peaked 3 days after the start of exposure in the brain (mean ± s.e. = 2.238 ± 0.342 ng g^−1^), liver (4.376 ± 0.537 ng g^−1^) and muscle (0.700 ± 0.050 ng g^−1^) (figure 3). Temazepam concentrations slowly decreased over time, with all samples being below the limit of quantification after 97 days of exposure. Further, oxazepam—a biologically active metabolite of temazepam—was also detected in trout tissues after exposure to temazepam. Similar to previous research in European perch [33], oxazepam accumulated at higher concentrations than its parent compound in the brains of trout. Specifically, tissue concentrations of oxazepam peaked 32 days after the start of exposure in the brain (6.058 ± 0.810 ng g^−1^), 17 days after the start of exposure in the liver (7.457 ± 0.858 ng g^−1^) and 22 days after the start of exposure in muscle (0.229 ± 0.085 ng g^−1^) (figure 3). The delayed peak in oxazepam concentrations may be owing to the time required for temperature-dependent biotransformation of temazepam into its metabolites in fish [33,57]. Importantly, levels of both temazepam and oxazepam detected in trout tissues in the current experiment are similar to those reported in other studies after environmentally realistic water-borne exposures [15], as well as blood plasma concentrations of oxazepam reported from wild European chub (*Squalius cephalus*; mean ± s.d. plasma concentration = 6.95 ± 7.82 ng ml^−1^) [58], suggesting that exposure levels in the current experiment were environmentally relevant.

## 4. Discussion

While much previous research has demonstrated that pharmaceutical pollution can affect the behaviour of aquatic organisms in the laboratory [6,11], whether exposure to these same pollutants can influence behaviour in the wild, and whether this has fitness consequences, is less clear. In a large, field-based study, we provide experimental evidence that exposure to environmentally realistic concentrations of the psychoactive pollutant temazepam can decrease lake migration success in brown trout. Given that downstream migration in adfluvial brown trout has important consequences for resource acquisition, growth and fecundity [22], disruption of this process may have fitness implications. This result highlights that pharmaceutical exposure could potentially reduce organismal fitness in the wild, with possible consequences for population persistence.

In line with predictions, brown trout smolts exposed to temazepam displayed decreased lake migration success relative to control groups. In particular, temazepam-exposed fish were less likely to reach the first downstream receiver in the River Ore relative to the control–no implant group, and were less likely to subsequently reach Lake Orsa compared with both of the control groups. Given that (i) tissue concentrations of temazepam were low and environmentally realistic, (ii) there was no effect of treatment on total time detected in the array, and (iii) there were temazepam-exposed fish that survived >154 days in both the field and laboratory experiments, such decreased lake migration success is highly unlikely to be owing to any directly toxic effect of temazepam in exposed smolts. Instead, there may be several reasons for reduced migration success in temazepam-exposed trout. First, brown trout exhibit substantial intraspecific diversity in migratory tendencies, with some individuals within a population opting to migrate while others remain resident in the river [22,59]. Indeed, temazepam-exposed smolts in the current study initially spent longer in the River Ore compared with the control–no implant group, suggesting that reduced lake migration success in exposed fish may partly be owing to treatment differences in migratory decisions. This result is in contrast with our prior predictions and previous research, which found that benzodiazepine exposure increased initial downstream river migration intensity over short distances in brown trout [16] and Atlantic salmon [28].

However, despite only *ca* 47% of temazepam-exposed fish that were detected in the river ultimately reaching Lake Orsa compared with *ca* 76% of control–implant fish, there was no difference between these groups in the initial time spent in the river. This suggests that the reduced lake migration success of exposed smolt is not likely to be explained by changes in migratory decisions alone, where exposed fish simply remain resident in the lower portion of the River Ore. Further, a hydropower dam without a fish passage was located *ca* 1.4 km upstream of the release site and prevented fish from moving upriver. We surmise that the river–lake confluence may have been a particularly dangerous area for trout smolts, with exposed fish potentially suffering higher predation rates than control fish. Indeed, prior work has found that northern pike predate heavily on brown trout smolts [60–62] and often aggregate at river mouths during the smolt emigration period [63]. The survival of migrating Atlantic salmon smolts is also often lowest when transitioning between rivers and lakes [63,64], highlighting the river–lake confluence as a particularly risky area for migrating salmonid smolts. Further, previous research has demonstrated that exposure to environmentally realistic concentrations of another anxiolytic drug—the benzodiazepine oxazepam—can increase risk-taking behaviour in fish [34,65,66]. Similar results have also been found for temazepam in European perch [15]. In this prior study, while only 50% of European perch in the control group left a refuge to explore a novel tank in laboratory assays, this increased to 80% and 94% for perch exposed to low (muscle concentration = 5.0 ± 3.8 ng g^−1^) and high (muscle concentration = 51.60 ± 19.1 ng g^−1^) concentrations of temazepam, respectively (although these effects were not statistically significant) [15]. However, similar effects of temazepam on risk-taking behaviour were not seen in sea-run brown trout during laboratory assays [16]. Nevertheless, together this research suggests that temazepam exposure may have increased the risk-taking behaviour of exposed brown trout smolts in the field during the current experiment, potentially resulting in high predation rates at the river–lake confluence. Future research using high-resolution acoustic telemetry [67] and predation sensor tags [64,68] will be needed to investigate whether fish exposed to pharmaceutical pollution utilize particular habitats or exhibit fine-scale movement patterns that make them more susceptible to predation in the wild.

It is also a possibility that, after reaching the first receiver in the River Ore, exposed trout returned upstream towards the release site and remained in the portion of the river below the hydroelectric power station (which has no fish passage solutions and, therefore, prevented movement further upstream). We expect that such an outcome could result in decreased fitness in exposed trout. In particular, the superior feeding opportunities in lakes results in lake-migrating trout often obtaining greater energy reserves, reaching a larger size at maturity, and achieving higher fecundity than conspecifics that remain within their natal river [22]. This suggests that any temazepam-exposed trout that returned upstream could potentially suffer decreased fitness. Further, temazepam-exposed trout may have also remained in the initial area of the river–lake confluence or in the margins of the lake where they were outside the range of acoustic receivers. As previously mentioned, river–lake confluences are known to be areas of high mortality for salmonid smolts, likely owing to aggregations of predatory fish [63,64]. Similarly, the initial section of Lake Orsa near the confluence with the River Ore, as well as the margins of the lake, are relatively shallow (*ca* 1−10 m). Prior research investigating predation probability by piscivorous birds in more than 25 000 salmonid smolts originating from the River Dal in central Sweden found that *ca* 32% of hatchery-reared brown trout smolts are consumed by predatory birds (e.g. great cormorants, *Phalarocorax carbo*) [69]. Thus, the shallow areas of the lake represent areas of high predation risk, highlighting that temazepam-exposed trout that remained within this initial section of Lake Orsa, or within the shallow margins of the lake, potentially suffered decreased survival. While our experimental design cannot distinguish between these scenarios (i.e. whether temazepam-exposed smolts were disproportionately predated upon at the river–lake confluence, remained within the margins of the lake, or returned upstream to remain resident within the River Ore), all three possibilities likely represent sub-optimal conditions for trout survival and/or growth, which may have consequences for individual fitness and population persistence.

For fish that reached Lake Orsa, we found that there were no treatment differences in the daily distance travelled throughout the study period, nor in the number of unique receivers visited by each fish per day. Previous work in the laboratory has found that fish exposed to environmentally realistic benzodiazepine concentrations often display increased swimming activity and movement [12,15,66] (but see [16,65]). Similar research has also demonstrated that exposure to dilute concentrations of other anxiolytic and antidepressant drug classes (e.g. selective serotonin reuptake inhibitors) can increase fish activity levels in the laboratory [70,71]. While there has been little experimental behavioural ecotoxicology research conducted in the field, a prior study found that European perch exposed to high concentrations (200 µg l^−1^) of the benzodiazepine oxazepam in the laboratory were more active and had larger home ranges once released into a small (*ca* 0.01 km^2^) lake [72]. However, similar results were not seen in a separate study on European perch after whole-lake exposure to oxazepam (11−24 µg l^−1^) [73]. Why movement rates and space use were not affected by temazepam exposure in the current study is not clear, but this suggests that care should be taken when extrapolating laboratory findings to exposed populations in the wild. However, we note that our measure of daily distance travelled is relatively coarse as it does not capture fine-scale movement dynamics. Given the dearth of field-based experimental behavioural ecotoxicology research, there is an urgent need for future work combining tools such as slow-release chemical implants and high-resolution acoustic telemetry to better understand whether psychoactive pharmaceuticals can influence movement rates and space use in the wild. It is also worth noting that there appeared to be an effect of the fat-based implant itself on some aspects of trout movement (e.g. initial time spent in the river) in the current study. The mechanisms driving this effect are not clear, but it will be important for future studies to utilize appropriate controls with sham implants (as was done in the current experiment) in order to isolate the effect of the pollutant itself on animal movement.

In conclusion, our research indicates that environmentally realistic concentrations of the psychoactive pollutant temazepam can influence movement dynamics and potentially affect fitness in brown trout—a species of high ecological and socio-economic value. Such effects may have implications for the persistence of migratory fish populations, and underscore the need to consider pharmaceutical pollution in our understanding of global change biology. What the long-term consequences of pollution-induced changes in movement dynamics are is not clear, especially when considering the substantial plasticity in salmonid migratory patterns [22] and previous research demonstrating the evolution of pollutant resistance in several fish populations [74,75]. Combining field-based behavioural ecotoxicology research that capitalizes on recent developments in high-resolution tracking [67,76,77] with long-term monitoring studies is needed to better understand the possible consequences that common neuroactive pollutants, such as pharmaceuticals, may have consequences for animal populations living in a rapidly changing world.

## Data Availability

All data and statistical code to reproduce the results are publicly available on the Open Science Framework online repository [78]. Supplementary material is available online [79].
